# Nitrite addition to acidified sludge significantly improves digestibility, toxic metal removal, dewaterability and pathogen reduction

**DOI:** 10.1038/srep39795

**Published:** 2016-12-22

**Authors:** Fangzhou Du, Jürg Keller, Zhiguo Yuan, Damien J. Batstone, Stefano Freguia, Ilje Pikaar

**Affiliations:** 1 The University of Queensland, Advanced Water Management Centre (AWMC), QLD 4072, Australia; 2 The University of Queensland, The School of Civil Engineering, QLD 4072, Australia

## Abstract

Sludge management is a major issue for water utilities globally. Poor digestibility and dewaterability are the main factors determining the cost for sludge management, whereas pathogen and toxic metal concentrations limit beneficial reuse. In this study, the effects of low level nitrite addition to acidified sludge to simultaneously enhance digestibility, toxic metal removal, dewaterability and pathogen reduction were investigated. Waste activated sludge (WAS) from a full-scale waste water treatment plant was treated at pH 2 with 10 mg NO_2_^−^-N/L for 5 h. Biochemical methane potential tests showed an increase in the methane production of 28%, corresponding to an improvement from 247 ± 8 L CH_4_/kg VS to 317 ± 1 L CH_4_/kg VS. The enhanced removal of toxic metals further increased the methane production by another 18% to 360 ± 6 L CH_4_/kg VS (a total increase of 46%). The solids content of dewatered sludge increased from 14.6 ± 1.4% in the control to 18.2 ± 0.8%. A 4-log reduction for both total coliforms and *E. coli* was achieved. Overall, this study highlights the potential of acidification with low level nitrite addition as an effective and simple method achieving multiple improvements in terms of sludge management.

Large amounts of waste activated sludge (WAS) are generated by wastewater treatment plants (WWTPs) worldwide^1^,^2^. The management of this sludge is a major issue for water utilities and comprises a significant fraction of the operational costs of WWTPs^3^. The main factors incurring these high operational costs are mainly related to the poor digestibility and dewaterability of WAS, whereas the presence of pathogens and toxic metals limits beneficial reuse. It also represents an environmental burden, especially when incineration or landfill is needed^4^.

Anaerobic digestion (AD) is a commonly used method for sludge stabilisation and energy recovery^5^. The relatively poor digestibility of WAS is a key process limitation, resulting in low biogas yields and long retention times^6^,^7^. Therefore, many studies aimed to enhance the digestibility of WAS using a variety of pre-treatment methods including biological, thermal, mechanical and chemical processes^5^. Although these methods have demonstrated enhancement of biogas production, the majority of these methods are relatively expensive or energy-intensive processes which have limited their widespread application. Therefore, there is a general interest and demand for more cost-effective and less energy-intensive treatment methods.

In addition to energy recovery, sludge can also be beneficially used as soil amendment or fertilizer^8^,^9^. However, the presence of toxic metals such as Cu and Zn limits the capacity of available land to receive sludge, particularly for repeated land applications. Consequently, the availability of arable land is becoming a limiting factor. Sludge with elevated toxic metal concentrations normally requires disposal/reuse in remote areas^10^, incurring higher transport costs. The disposal and transport costs are also strongly affected by the poor dewaterability of sewage sludge^11^.

It was reported that the treatment by free nitrous acid (FNA) significantly enhanced both anaerobic and aerobic digestion^12^,^13^ of WAS at an FNA concentration of only 2 mg FNA-N/L (achieved by dosing up to 1500 mg NaNO_2_/L at pH 5.5). Moreover, considering FNA was reported for its ability of cellular destruction and the strong biocidal effect^14^, FNA treatment may thus be effective in reducing pathogen concentrations in WAS. The latter is required as a stabilisation process before land application^15^. Very recently, it was discovered that sludge acidification to pH 2 combined with the addition of only 10 mg NO_2_^−^-N/L strongly enhances toxic metal removal by solubilising organically bound metals^16^. It was assumed that the latter was caused by the disruption of extracellular polymeric substances (EPS) caused by FNA treatment. This hypothesis is supported by a very recent study which showed that FNA directly affects EPS by specifically targeting protein-like substances and hence breaking down the macromolecules within the EPS matrix into smaller molecules^17^. Toxic metals, including Cu and Zn, are also known for their inhibitory effect on the AD process^18^,^19^. The enhanced removal of toxic metals may thus further improve the AD of WAS. It is known that EPS plays an important role in the dewaterability of sludge^20^, and as such the disruption of EPS by FNA may also affect the dewaterability of WAS.

Considering the above-mentioned recent studies outlining the versatile characteristics of FNA, we hypothesize that by the addition of low levels of nitrite to acidified WAS, it would be possible to simultaneously achieve considerable improvements in digestibility, toxic metal removal, dewaterability and pathogen reduction in one simple treatment step. The possibility of achieving multiple benefits in sludge management using one simple treatment method would make the proposed method attractive for practical application. This study therefore aims to investigate the effects of sludge acidification with the addition of small amounts of nitrite on the methane production from anaerobic digestion, dewaterability and pathogen reduction. In addition, this study also aims to determine the effect of enhanced toxic metal removal on the sludge digestibility. Model based analysis was adopted to estimate the methane production during continuous anaerobic digestion at a hydraulic retention time of 20 days.

## Results and Discussion

### Effect of acid and nitrite addition on sludge digestibility

**Figure 1:**
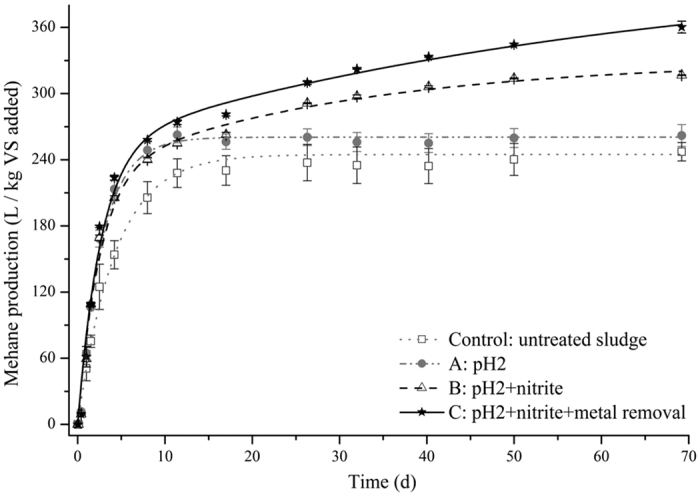
Cumulative methane production from WAS exposed to different pre-treatment. Dots represent the experimental data and lines represent the model fitting. Control and treatment (**A**) were fitted using first-order one-substrate model, Treatment (**B,C**) were fitted using first-order two-substrate model. Error bars show standard deviation from triplicate tests.

The cumulative methane production from the anaerobic digestion of WAS exposed different treatments is shown in Fig. 1. At day 69 of BMP tests, the acidification treatment at pH 2 (treatment A) only resulted in a minor increase in methane production of 5.9%. On the other hand, the treatment at pH 2 with nitrite addition (treatment B) resulted in an increase in the methane production of 28.0%. A further enhancement was observed for treatment C (pH 2 with nitrite addition and toxic metal removal), achieving an increase in methane production of 45.7%. The results obtained here clearly show the effect of nitrite addition under acidic conditions (*i.e.* pH = 2) as well as the importance of toxic metal removal on the methane production.

The hydrolysis rate coefficient (*k*) and biochemical methane potential (*B*_*0*_) were estimated using both first-order one-substrate model and two-substrate model. The one-substrate model gave satisfactory fit to the experimental data of control and treatment A (pH 2). There was insufficient evidence (*p* > 0.05) to support a two-substrate model for control and treatment A (*i.e.* pH 2). However, nitrite addition (treatments B and C) had strong evidence (*p* < 0.05) of a two-substrate system with fits shown in Fig. 1. The *k* of treatment A was estimated to be 0.37 ± 0.05 d^−1^, higher than the estimated *k* of the control group with a value of 0.26 ± 0.02 d^−1^ (Table 1). The *k*_*rapid*_ of treatments B and C were estimated to be around 0.4 d^−1^.

In practice, mesophilic AD is more likely to be adopted in continuous mode with a typical HRT of about 20 days^5^. Therefore, a continuous reactor model with the obtained *k* and *B*_*0*_ was adopted to estimate the cumulative methane production for a continuous anaerobic digester operating at a hydraulic retention time (HRT) of 20 days (Table 1). By conducting two-tailed *t*-tests, it was found that the difference in methane production from each treatment (control, A, B and C) was significant (*p* < 0.01) to all the other treatments.

**Figure 2:**
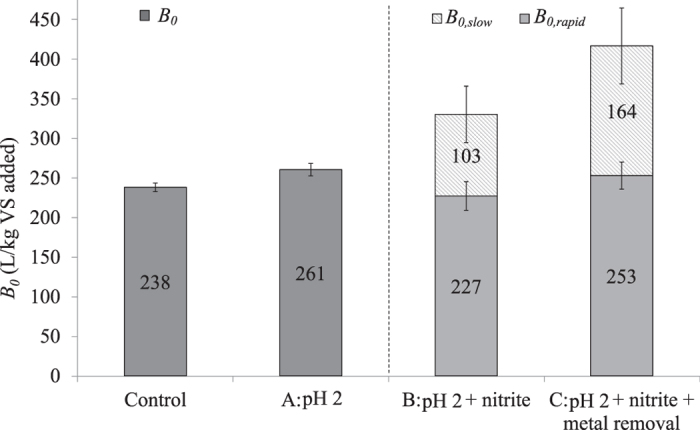
Estimated biochemical methane potential (*B*_*0*_) of WAS after different treatments. The *B*_*0*_ of Control and treatment (**A**) were estimated using the one-substrate model. The *B*_*0*_of treatment (**C**,**D**) were estimated using the two-substrate model with *k*_*slow*_.at their optima. The top error bars for treatment (**B**,**C**) represents the combined error (95% confidence interval) of *B*_*0,rapid*_ + *B*_*0,slow*_.

The *B*_*0*_ values for all the treatments are presented in Fig. 2. As discussed above, treatments B and C were fitted with a two-substrate system. So the values of *B*_*0*_ for treatment B and C were composed of *B*_*0,rapid*_and *B*_*0,slow*_. The error in the two substrate model was based on the error in methane yields for rapid and slow substrates, with hydrolysis coefficients fixed to their optima. The *B*_*0,rapid*_ of treatment B and C reached comparable values to the *B*_*0*_ of the control and treatment A. The increase of the final methane production mainly came from the addition of the slowly degradable substrates. In our previous study, nitrite addition to acidified sludge released organically bound metals in sludge^16^. Since FNA directly affects EPS by specifically targeting protein-like substances therby breaking down the macromolecules within the EPS matrix into smaller molecules^17^ and the fact that EPS is known to be relatively resistant to AD^5^, the disrupted EPS might be converted from non-digestible to (slowly) digestible. This is supported by the observed improvement of methane production in this study.

FNA has been reported for its ability to increase the biodegradability of sludge in both aerobic^13^ and anaerobic^12^ conditions, in which sludge treatment at 50–300 mg NO_2_^−^-N/L and pH 5.5 resulted in higher SCOD concentrations compared to pH 5.5 treatment. It was speculated that the higher SCOD subsequently resulted in higher *k* and *B*_*0*_values during the BMP test. However in this study, the treatment at pH 2 with nitrite addition did not cause additional increase of SCOD of treated WAS or *k* (or *k*_*rapid*_), compared to pH 2 only treatment. The increase in total methane production and *B*_*0*_ revealed that nitrite addition at pH 2 converted part of non-degradable substrates into slowly degradable substrates in the insoluble fraction of WAS. Hence, the results obtained in this study may indicate that the effectiveness of nitrite at pH 2 is based on a different working mechanism than at pH 5.5. Further research is warranted to reveal the nature of the effect of nitrite on sludge at pH 2.

Toxic metals are known for their inhibition effect on AD process, even when their concentration is lower than 10 mg/L^21^. The initial concentrations of Cu and Zn in this study were 10.9 ± 0.6 mg/L and 16.1 ± 0.9 mg/L, respectively. Similar to our previous study^16^, the addition of nitrite substantially enhanced the toxic metal removal, achieving 43% and 70% for the removal efficiency of Cu and Zn, respectively (see supplementary Table S1). By subsequent solid/liquid separation and precipitation, toxic metals were removed from the sludge in treatment C, achieving 294 ± 8 mg/kg dry solids and 207 ± 8 mg/kg dry solids for Cu and Zn as the final concentrations, respectively. The enhanced removal of toxic metals significantly (*p* < 0.05) further increased cumulative methane production by 18% as presented in Fig. 1. It was suspected that the partial release of inhibitory impact of Cu and Zn on the AD process was the reason for this increase of methane production.

### Sludge dewaterability

**Figure 3:**
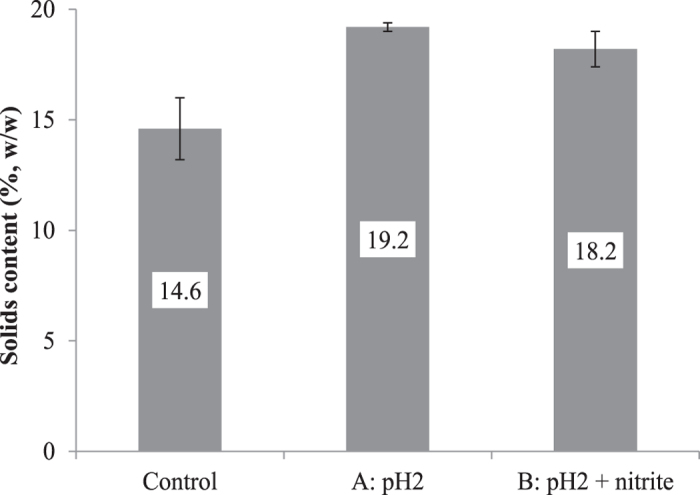
Solids content of belt filter dewatered sludge from different treatments.

Figure 3 shows that the treatment at pH 2, either with or without nitrite addition, resulted in a significant increase (*p* < 0.05) in the solids content of the dewatered sludge cake. It can also be seen that the sludge dewaterability was not further improved by the nitrite addition, indicating that the enhanced dewaterability was caused by the acidic conditions. This is in agreement with Chen *et al*.^22^ who found that acidification of sludge to pH 2.5 increased the solids content from about 18% to 24% through filtration dewatering. As a result of the improved sludge dewaterability, the amount of wet sludge to be disposed would be reduced by about 20%.

### Pathogen reduction

**Figure 4:**
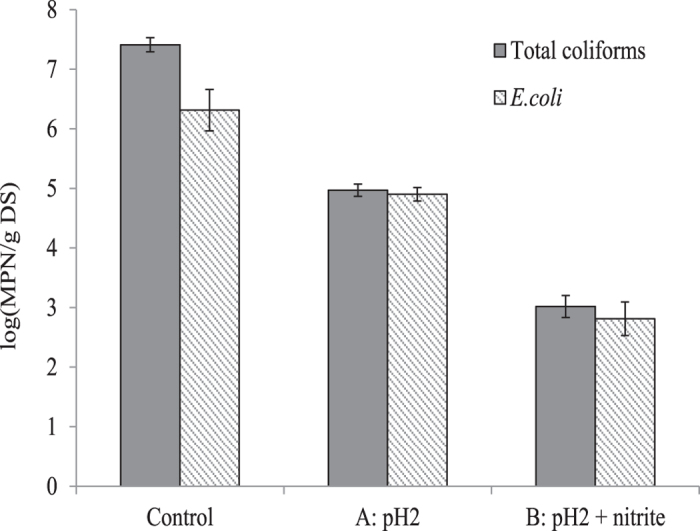
Concentration of total coliforms and *E. coli* in undigested sludge after different treatments.

Figure 4 shows the impact of acidification with and without nitrite addition on the pathogen reduction. It can be seen that the treatment at pH 2 with nitrite addition resulted in a significantly (*p* < 0.05) higher reduction in pathogen indicators compared to acidification alone, achieving a 4 log reduction for both total coliforms and *E. coli* in terms of the most probable number (MPN) per gram of dry solids (DS), compared to a 3 and 2 log units of concentration abatement for total coliforms and *E. coli*, respectively.

Regulation requires that WAS is stabilized to a specific level (*E. coli* < 100 MPN/g DS and Faecal coliforms < 1000 MPN/g DS) to achieve Grade A stabilisation for unrestricted land application^10^. In this study, the combination of pH 2 and nitrite achieved a 4 log unit reduction for MPN concentration of total coliforms and *E. coli*. In a study of Gantzer *et al*.^23^, it was found that mesophilic anaerobic digestion resulted in a 2 log reduction in MPN. While this study cannot be directly compared with the results obtained here since sludge from a different origin was used, it does clearly show that FNA is an effective method for pathogen reduction. Equally important, the FNA based treatment employed in this study only required a HRT of 5 h, much shorter than conventional mesophilic AD (commonly around 20 days)^5^. However, the treatment by acidification with nitrite only achieved Grade A threshold for faecal coliforms while still one order of magnitude away from the threshold for *E. coli.* Further pathogen reduction may be achieved by increasing the nitrite dosing and HRT.

### Implications for practice

Here, it was demonstrated that sludge acidification with the addition of only 10 mg/L of NO_2_-N simultaneously enhances sludge digestibility, toxic metal removal, dewaterability and pathogen reduction. Recently, the feasibility of on-site production of acid and caustic using a novel three compartment electrochemical cell was demonstrated^24^. In a practical situation, the increase in biogas production could be used to drive the electrochemical process and as such could potentially allow for the energy-neutral generation of acid and caustic. In addition, FNA could also be produced on-site directly from anaerobic digester liquor^25^. Combining these two recent findings could potentially allow for an energy-neutral sludge management strategy without the need for external chemical input.

## Methods

### Sludge source

Waste activated sludge (WAS) was collected from Luggage Point WWTP (Brisbane, Queensland, Australia). After collection, the sludge was immediately stored at 4 °C, and all experiments were started within two weeks of sludge collection. The main sludge characteristics were: total solids (TS) 21.3 ± 0.1 g/L; volatile solids (VS) 17.3 ± 0.1 g/L; total chemical oxygen demand (TCOD) 28.0 ± 0.1 g/L; soluble chemical oxygen demand (SCOD) 0.84 ± 0.10 g/L, and pH 6.4 ± 0.1. The inoculum for the BMP tests was collected from the mesophilic anaerobic digester at the same WWTP treating a mixture of WAS and primary sludge. The TS and VS content of the inoculum sludge were 24.6 ± 0.2 g/L and 16.8 ± 0.0 g/L, respectively. All sludge characteristics were analysed in triplicate.

### Sludge treatment

To investigate the impact of acidification and nitrite treatment on the sludge digestibility, dewaterability and pathogen reduction, 3 different treatment conditions were investigated and compared with untreated sludge (control group). In treatment A, WAS was acidified to pH 2 for a period of 5 hours. In treatment B and C, WAS was acidified to pH 2 with the addition of 10 mg NO_2_^−^-N/L for a period of 5 hours. In treatment C, the acidification step was followed by a metal removal step. Metals were removed using the following procedure. Firstly, the WAS was centrifuged at 13,000 × g for 20 min (Centrifuge 5810 R, Eppendorf, Germany) to separate the solids and the WAS liquor. The WAS liquor was subsequently neutralized to pH 7 to precipitate the metals. The precipitated metals were removed from the solution by filtration using Millipore filters (0.22 μm pore size, Millipore Express, USA). Subsequently, the filtrate and WAS solids were mixed (*i.e.* sludge minus metals removed).

In all experiments, the acidification and neutralisation was done by addition of 1 M HCl and 1 M NaOH respectively. A volume of 500 mL of WAS was used for each of the treatment conditions. All sludge treatment experiments were conducted in triplicate. For each triplicate, the treated sludge was subsequently used in 3 series of experiments to determine their impact on the sludge digestibility, dewaterability and pathogen reduction.

### Biochemical Methane Potential (BMP)

BMP tests were adopted to investigate the sludge digestibility as described by Jensen *et al*.^26^. For each BMP test, 50 mL of inoculum was used. Substrate (*i.e.* WAS from the control group and each treatment conditions) was added at an inoculum to substrate ratio of 2.0 on a VS basis. The volume of each BMP vial was topped up with Milli-Q water to 80 mL. A blank was set up by mixing 50 mL of inoculum and 30 mL of Milli-Q water. After treatments A, B and C, the treated sludge was neutralized to its initial pH (*i.e.* 6.5 ± 0.3) before mixing with inoculum. To eliminate the possibility that results were affected by the shearing force during the centrifugation step in treatment C, the samples from control as well as treatments A and B were also centrifuged and mixed under identical condition (but without removing the metals) prior to BMP tests.

After filling with inoculum and substrate, the vials were flushed with nitrogen gas for 30 seconds at a flow rate of 3.5 L/min and then sealed with a butyl rubber stopper retained with an aluminum crimp-cap. An incubator maintaining 37 ± 1 °C was used to store all the testing vials. The BMP tests were terminated after 70 days. The biogas (CH_4_, H_2_ and CO_2_) production was measured every 1–2 days over the first 10 days and every 4–6 days afterwards. The biogas production from WAS was obtained by subtracting the average biogas production from the blank group (see supplementary Fig. S1). All tests were conducted in triplicates.

The apparent first order hydrolysis rate coefficient (*k*) and biochemical methane potential (*B*_*0*_) were adopted as the key parameters for the assessment of WAS digestibility after different treatment. The values for them were estimated by fitting BMP data to a first-order kinetic model using a modified version of Aquasim 2.1d with sum of squared errors (*J*_*opt*_) as an objective function, as described by Batstone *et al*.^27^ and Jensen *et al*.^26^. The uncertainty surfaces of *B*_*0*_ and *k* were estimated based on a model-validity *F*-test with 95% confidence limits. A one-substrate model and a two-substrate model^12^ were used. The one-substrate model is described in Eq. (1):


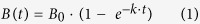


where *B(t)* is cumulative methane yield at time t (L CH_4_/kg VS added); *B*_*0*_ is the biochemical methane potential; *k* is hydrolysis rate coefficient (d^−1^); *t* is time (d). In the two-substrate model, two types of substrates, *i.e.* rapidly degradable substrate and slowly degradable substrate, were considered, see Eq. (2):


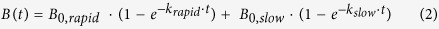


where *B(t)* is cumulative methane yield at time *t*; *B*_*0,rapid*_ is the biochemical methane potential of rapidly degradable substrate; *k*_*rapid*_ is hydrolysis rate coefficient of rapidly degradable substrate; *B*_*0,slow*_ is the biochemical methane potential of slowly degradable substrate; *k*_*slow*_ is hydrolysis rate coefficient of slowly degradable substrate; *t* is time. Total biochemical methane potential *B*_*0*_ = *B*_*0,rapid*_ + *B*_*0,slow*_. Model fitness was evaluated by ANOVA by an *F-*test on non-linear regression sum of squared errors to assess whether Eq. (2) was significantly better.

To estimate the impact of the applied pre-treatment on methane production in a practical situation, *i.e.* continuous anaerobic digestion with a hydraulic retention time (HRT) of 20 days, a continuous reactor model was adopted with the *k* and *B*_*0*_ gained from the previous steps. A single substrate assumption was applied to predict the performance at an HRT of 20 days due to the relatively high uncertainty (and correlated parameter space) that existed with the two-substrate model. This is justified as the single substrate model provides a reasonable prediction of continuous performance specifically at a 20 day point. Numerical uncertainty propagation method described by Batstone^28^ was used to estimate the methane production, using the following equation:

One-substrate model:


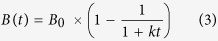


where *B(t)* is cumulative methane yield at time *t* (L CH_4_/kg VS added); *B*_*0*_ (*B*_*0,rapid*_and *B*_*0,slow*_) is biochemical methane potential (for rapidly and slowly degradable substrate, respectively); *k (k*_*rapid*_ and *k*_*slow*_) is hydrolysis rate coefficient (for rapidly and slowly degradable substrate, respectively) (d^−1^); *t* is time (d). *t* = 20 d in this case. According to Batstone^28^, the calculation of *B(t)* using Equation 3 was conducted by 10,000 simulations, applying observed distributions for the parameters. The average values of the *B(20*) were adopted as the estimated practical methane production in a continuous anaerobic digester with 20 d HRT.

Through AD, the mass of sludge is reduced due to the degradation of VS into methane. It was assumed that the inorganic solids in WAS would not be digested. The VS destruction can be calculated in accordance with Eq. (4):


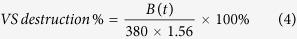


where *B(t)* is the biochemical methane production at time *t* (L CH_4_/kg VS added); *380* is theoretical biochemical methane potential under standard conditions (25 °C, 1 atm) (L CH_4_/kg TCOD); *1.56* is the TCOD/VS ratio of the WAS used in this study.

### Dewaterability

The impact of treatments A and B on the dewaterability of the treated sludge was evaluated by the belt filter proxy test described by Higgins *et al*.^29^ without using any conditioning agent. The sludge dewaterability was determined and compared by measuring the dewatered sludge cake solids concentration (% w/w).

### Pathogen reduction

Total coliforms and *E. coli* were selected as indicators of pathogen in WAS^10^,^30^. The impact of the different sludge treatment methods on the pathogen reduction were analyzed using the Colilert^®^-18 kit test (IDEXX, U.S.A), according to Eccles *et al*.^31^. Prior to analysis, the WAS subject to treatments A and B was neutralized to their initial pH values of 6.5 ± 0.3.

### Analytical methods

The TS and VS concentrations were determined using standard methods^32^. TCOD and SCOD were analysed using Spectroquant^®^ COD cell tests (Merck, Germany). For analysis of the SCOD concentrations, samples were first filtered using a 0.22 μm syringe filter (Milipore, USA). In all experiments, pH was measured using a handheld pH meter (pH meter 11 series, Oakton, Australia). The analysis of toxic metal concentrations was conducted by inductively coupled plasma optical emission spectrometry (ICP-OES, 7300DV, PerkinElmer, USA) according to Du *et al*.^16^.

During the course of the BMP experiments, the gas phase pressure was measured by a monometer before each sampling event. The cumulative volume of the produced gas was calculated according to the pressure in the headspace (80 mL) and expressed under standard conditions (25 °C, 1 atm). The biogas composition (CH_4_, CO_2_, H_2_) was analysed using a gas chromatograph (GC-2014, Shimadzu, Australia) equipped with a thermal conductivity detector and a HAYESEP Q 80/100 packed column, as described by Astals *et al*.^33^. Argon was used as a carrier gas with a flow rate of 28 mL/min at 135.7 kPa. The temperature of the chromatograph injector was set at 348.15 K, whereas the temperature of the oven and detector were set at 318.15 K and 373.15 K, respectively.

To evaluate the impacts of the treatments, measures of average sludge characteristics after treatment were compared with the control group and each other, using 95% confidence interval in mean based on a two-tailed t-test in measure standard error, according to Batstone (2013)^28^.

## Additional Information

**How to cite this article**: Du, F. *et al*. Nitrite addition to acidified sludge significantly improves digestibility, toxic metal removal, dewaterability and pathogen reduction. *Sci. Rep.* **6**, 39795; doi: 10.1038/srep39795 (2016).

**Publisher's note:** Springer Nature remains neutral with regard to jurisdictional claims in published maps and institutional affiliations.

## Supplementary Material

Supplementary Information

## Figures and Tables

**Table 1:** Sludge solubilisation after different treatment, VS destruction and estimated hydrolysis rate coefficient (*k*) for BMP tests.

Sludge treatment groups	SCOD (mg/L)^a^	VS destruction after 69 d of BMP^a^	Hydrolysis rate coefficient (*k* or *k*_*rapid*_, d^−1^)	Estimated CH_4_ production from continuous AD with 20 d HRT^d^ (L/kg VS)
**Control**	842 ± 100	42 ± 1%	0.26 ± 0.02^b^	200 ± 6
**A: pH2**	1950 ± 33	44 ± 2%	0.37 ± 0.05^b^	230 ± 8
**B: pH2 + nitrite**	1749 ± 17	53 ± 0%	0.41 ± 0.07^c^	247 ± 16
**C: pH2 + nitrite + metal removal**		61 ± 1%	0.37 ± 0.06^c^	267 ± 20

^a^Error estimates are standard deviation from triplicate tests.

^b^Estimated from one-substrate model with 95% confidence interval based on a two-tailed t-test in parameter standard error.

^c^Estimated from two-substrate model with 95% confidence interval based on a two-tailed t-test in parameter standard error.

^d^Estimated according to Batstone^28^ with 95% confidence interval based on a two-tailed t-test in parameter standard error.
