# Supplementary material for: Nitrite addition to acidified sludge significantly improves digestibility, toxic metal removal, dewaterability and pathogen reduction
Source: Sci Rep. 2016 Dec 22;6:39795. doi: 10.1038/srep39795 (PMC5177897; doi:10.1038/srep39795)
Supplement: Supplementary Information [file srep39795-s1.pdf]

Supplementary information

Nitrite addition to acidified sludge significantly improves digestibility, toxic metal removal, dewaterability and pathogen reduction

Authors: Fangzhou Du[1], Jürg Keller[1], Zhiguo Yuan[1], Damien J. Batstone[1], Stefano Freguia[1], Ilje Pikaar[1,2*]

[1]*The University of Queensland, Advanced Water Management Centre (AWMC), QLD 4072, Australia*

[2]*The University of Queensland, The School of Civil Engineering, QLD 4072, Australia*

**Correspondence should be addressed to:*

Ilje Pikaar, The School of Civil Engineering, The University of Queensland, St. Lucia, QLD 4072, Australia

Phone: +61 7 33465 1389; E-mail: i.pikaar@ uq.edu.au

Number of pages: 2

Number of figures: 1

Number of tables: 1

Table S1 Solid phase Cu and Zn concentrations

	Cu (mg/kg DS)[a]	Zn (mg/kg DS) [a]
Control	518 ± 29	768 ± 45
After HCl treatment (pH 2)	397 ± 37	276 ± 27
After HCl + nitrite treatment (pH 2 and 10 mg/L N-NO_2^-)	294 ± 8	207 ± 8
Grade A biosolids threshold[b]	100	200
Grade B biosolids threshold[b]	375	700
Grade C biosolids threshold[b]	2000	2500

Note: a. All the tests were conducted in triplicate.

b. Based on NSW-EPA[1].

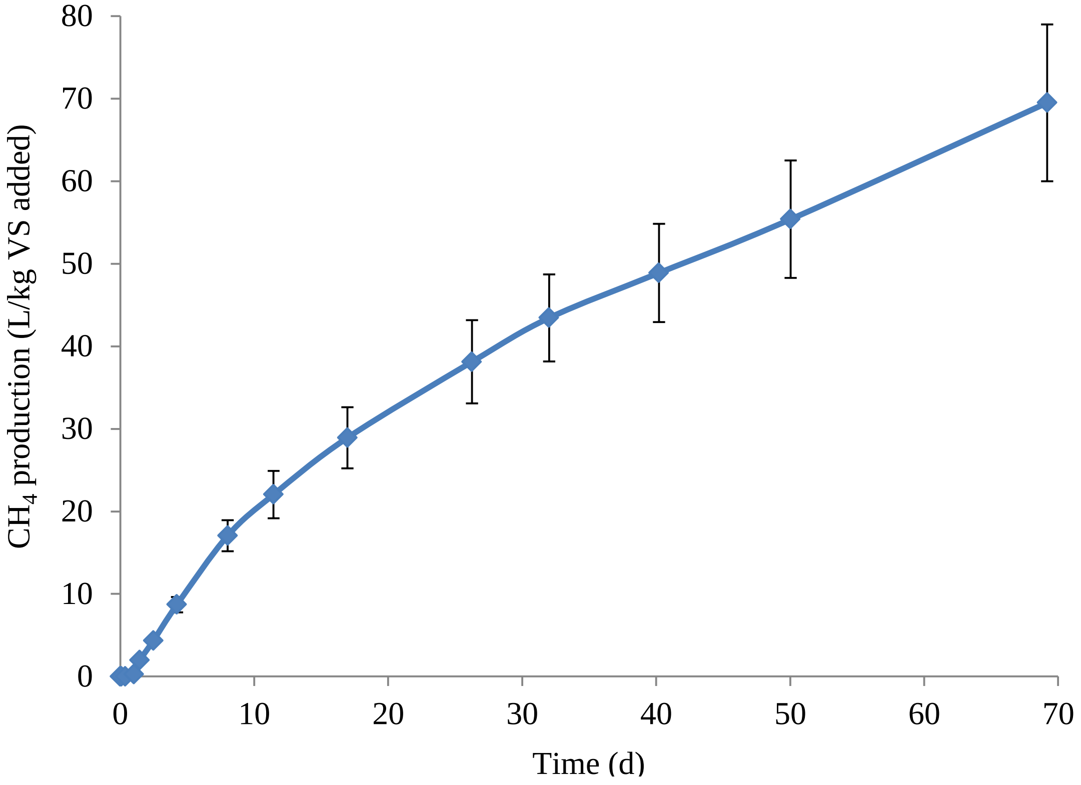

Figure S1 The bio-methane production during the BMP tests in the blank group over a period of 68 days (n=3).

References

1 NSW-EPA. *Environmental guidelines: use and disposal of biosolids products*. (2000).
